# mTORC1 as a Regulator of Mitochondrial Functions and a Therapeutic Target in Cancer

**DOI:** 10.3389/fonc.2019.01373

**Published:** 2019-12-13

**Authors:** Karen Griselda de la Cruz López, Mariel Esperanza Toledo Guzmán, Elizabeth Ortiz Sánchez, Alejandro García Carrancá

**Affiliations:** ^1^Posgrado en Ciencias Biomédicas, Instituto de Investigaciones Biomédicas, Universidad Nacional Autónoma de México, Mexico City, Mexico; ^2^División de Investigación Básica, Instituto Nacional de Cancerología, Mexico City, Mexico; ^3^Unidad de Investigación Biomédica en Cáncer, Instituto de Investigaciones Biomédicas, Universidad Nacional Autónoma de México & Instituto Nacional de Cancerología, Secretaría de Salud, Mexico City, Mexico

**Keywords:** mTORC1, mitochondria, mitochondrial functions, cancer, therapy

## Abstract

Continuous proliferation of tumor cells requires constant adaptations of energy metabolism to rapidly fuel cell growth and division. This energetic adaptation often comprises deregulated glucose uptake and lactate production in the presence of oxygen, a process known as the “Warburg effect.” For many years it was thought that the Warburg effect was a result of mitochondrial damage, however, unlike this proposal tumor cell mitochondria maintain their functionality, and is essential for integrating a variety of signals and adapting the metabolic activity of the tumor cell. The mammalian/mechanistic target of rapamycin complex 1 (mTORC1) is a master regulator of numerous cellular processes implicated in proliferation, metabolism, and cell growth. mTORC1 controls cellular metabolism mainly by regulating the translation and transcription of metabolic genes, such as peroxisome proliferator activated receptor γ coactivator-1 α (PGC-1α), sterol regulatory element-binding protein 1/2 (SREBP1/2), and hypoxia inducible factor-1 α (HIF-1α). Interestingly it has been shown that mTORC1 regulates mitochondrial metabolism, thus representing an important regulator in mitochondrial function. Here we present an overview on the role of mTORC1 in the regulation of mitochondrial functions in cancer, considering new evidences showing that mTORC1 regulates the translation of nucleus-encoded mitochondrial mRNAs that result in an increased ATP mitochondrial production. Moreover, we discuss the relationship between mTORC1 and glutaminolysis, as well as mitochondrial metabolites. In addition, mitochondrial fission processes regulated by mTORC1 and its impact on cancer are discussed. Finally, we also review the therapeutic efficacy of mTORC1 inhibitors in cancer treatments, considering its use in combination with other drugs, with particular focus on cellular metabolism inhibitors, that could help improve their anti neoplastic effect and eliminate cancer cells in patients.

## Introduction

Cellular metabolism involves a set of highly coordinated activities in which numerous enzymes collaborate to convert nutrients into building blocks toward generation of macromolecules, energy, and cellular biomass. In cancer, genetic, and epigenetic changes can disturb key enzymes or rewire oncogenic pathways, resulting in cell metabolism alterations (1). In 1924 Otto Warburg observed that tumor cells prefer aerobic glycolysis to generate ATP and lactate even in presence of oxygen, process known as the “Warburg effect” (2). For a long time it was believed that this preference for the Warburg effect was due to a failure in the mitochondrial function. Nevertheless, in recent years, there were significant progresses in our understanding of metabolic regulation in cancer and contrariwise, it was demonstrated that cancer cells have a functional mitochondrion. Furthermore, it was shown that oxidative phosphorylation (OXPHOS) is crucial for ATP production and tumor progression (3). However, mitochondria perform many functions beyond energetic production, including generation of redox molecules and the regulation of cell signaling, cell death, biosynthetic metabolism, and generation of reactive oxygen species (ROS) (4).

Mitochondrial ROS are the byproducts of metabolic processes during which electrons escape from the mitochondrial electron transport chain and then are captured by molecular oxygen to generate superoxide anions (${\text{O}}_{2}^{-}$) (5). Mitochondrial ROS exhibit both, a tumor promoting or tumor suppressing roles, depending on their levels and their oxidative potential. ROS are highly reactive species that produce oxidized proteins, lipids and nucleic acids, either behaving as damaging or as signaling species in cell metabolism. For instance, low levels of ROS have a pronounced proliferative effect but high levels induce tissue damage and consequently cell death (6). Despite the potential damaging roles of high ROS, cancer cells posses ROS-scavenging systems aimed to maintain ROS homeostasis, being the two major players Glutathione (GSH) and Thioredoxin (Txn) (7). Mitochondrial functions confer high levels of cellular plasticity, which permits a fast adaptation to challenging microenvironments conditions, such as hypoxia and nutrient deficiency, two very common consequences in tumors (8). On the other hand, accumulation of damaged mitochondria can be dangerous to cells; mitochondrial quality and quantity are processes severely monitored to ensure balance in cell physiology (9). Damaged or unwanted mitochondria can be selectively removed by mitophagy, a lysosome-dependent catabolic degradation process (10). Mitochondrial functions are matched by their morphological and structural changes, during the lifetime of a cell, the mitochondrial homeostasis network is constantly shaped by fission and fusion events (11).

In the process of tumor initiation and progression, cancer cells are exposed to harsh condition such as hypoxia or nutrient depletion in the tumor microenvironment. To survive in this severe environment, cancer cells must sense, and respond to the status of nutrient availability in the extracellular environment. The cell has several nutrients sensors responsible for maintaining cell homeostasis with the extracellular environment, such as the mammalian/mechanistic target of rapamycin complex 1 (mTORC1) that drives ATP-consuming cellular processes (anabolic) necessaries for cellular proliferation and growth (12). Another important sensor is the serine/threonine kinase AMP-activated protein kinase (AMPK), which, as its name implies, senses cellular AMP levels and coordinate a metabolic switch from anabolism toward catabolism under energy deprivation conditions such as hypoxia and hypoglycemia (13). AMPK has a wide variety of cell targets, one of which is mTORC1. AMPK activation suppresses mTORC1 signaling, thus regulating energy metabolism by stimulating the activity of several transcriptional controllers of metabolic enzymes such as peroxisome proliferator activated receptor γ coactivator-1 α (PGC-1α), sterol regulatory element-binding protein 1/2 (SREBP1/2), and hypoxia inducible factor-1 α (HIF-1α) (14). Interestingly, has been shown that mTORC1 also regulates mitochondrial oxidative metabolism (15–17). Moreover, mitochondrial oxidative metabolism is a very important mechanism for cancer development, acquired resistance against chemotherapy, and increased hypoxia tolerance in tumor microenvironment.

In this review we explain the participation of mTORC1 in the regulation of mitochondrial ATP producing capacity and we discuss how this process affects tumor cells. On the other hand, the mitochondrial function is directly associated with mitochondrial morphology regulated through fusion and fission processes thus, we review the current knowledge about the relationship of mitochondrial morphology highlighting mTORC1 participation in cancer. On the other hand it is known that glutamine, the most abundant free amino acid in blood, is uptaked by tumor cells and converted into α-ketoglutarate (α-KG) that fuels the tricarboxilic acid (TCA) cycle and OXPHOS in tumor mitochondrial. Therefore, we discuss how glutamine and mTORC1 participate in tumor development. Additionally, it was shown that mutations in nuclear and mitochondrial DNA lead to deregulation of important metabolic enzymes promoting the accumulation of intermediary metabolites, known as oncometabolites which in turn support cancer development. In this review, we depict the role of mTORC1 in the regulation of oncometabolites, as well as the therapeutic efficacy of mTORC1 inhibitors in cancer treatment.

## Structure and Functions of mTORC1

The protein serine threonine kinase TOR (target of rapamycin) was initially identified in *Saccharomyces cerevisiae* as a target of the macrolide fungicide rapamycin and later, the mammalian counterpart was identified and named mammalian/mechanistic target of rapamycin (mTOR), also known as FRAP (FKBP12-rapamycin-associated protein), RAFT (rapamycin and FKB12 target), and RAPT1 (rapamycin target 1) (18, 19). TOR is a large (~280 kDa) serine/threonine protein kinase that belongs to the family of phosphoinositide 3-kinase (PI3K)-related kinase (20). The mTOR protein interacts with other proteins and form two distinct multiprotein complexes: mTOR Complex 1 (mTORC1) and mTOR Complex 2 (mTORC2), either one with a different sensitivities to rapamycin (21). mTORC1 is inhibited by rapamycin, while mTORC2 is resistant to acute rapamycin treatment, however in some types of cells (HeLa and PC3) this mTORC2 complex can be inhibited by longer rapamycin treatments (over 24 h) (22).

**Figure 1:**
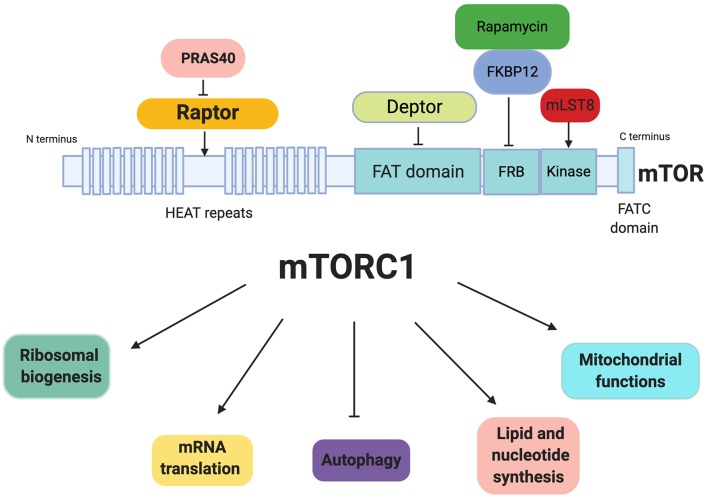
Mechanistic target of rapamycin complex 1 (mTORC1) and regulation of cellular processes. mTORC1 is a complex with DEPTOR and PRAS40 as negative regulators and RAPTOR and mSLT8 as positive regulators. Rapamycin-FKBP12 inhibits the mTOR kinase by directly blocking substrates recruitment and by further restricting active-site access. mTORC1 regulates different cellular processes such as ribosomal biogenesis, mRNA translation, autophagy, lipid and nucleotide synthesis, and mitochondrial functions.

mTORC1 is composed by the regulatory-associated protein of mTOR (Raptor), a scaffolding protein important for mTORC1 assembly, stability, substrate specificity and regulation (23), and by the proline-rich AKT substrate 40 kDa factor protein (PRAS40) (24), that associates with Raptor and inhibits mTORC1 activity. mTORC2 complex is composed by the rapamycin-insensitive companion (Rictor) (25), a component essential for both, complex formation, and their biological function, the mammalian stress-activated map kinase-interacting protein 1 (mSin1) an essential component required for complex formation and kinase activity (26), and by Protor 1 (Protein observed with Rictor 1), required to allow efficient regulation of mTORC2 targets (27). Both mTORC1 and mTORC2 are composed by mTOR protein, a mammalian lethal with sec13 protein 8 (mLST8, also known as GβL), DEP domain-containing mTOR interacting protein (DEPTOR), and Tel two interacting protein 1 (TTI1/TEL2) complex. mLST8 is associated with the catalytic domain of mTOR and may stabilize the kinase activation loop, DEPTOR on the contrary inhibits mTOR activity, TTI1/TEL2 is a mTOR interacting protein important for mTOR stability and assembly of the mTOR complex and maintain their activities (28) (Figure 1).

mTORC1 is activated via growth factors stimulation [epidermal growth factor (EGF), insulin-like growth factor (IGF)], increase in amino acid levels such as leucin and arginine and cellular energy status (29–31), promoting protein and lipid synthesis, as well as ribosome biogenesis impacting on cell proliferation and growth, autophagy and metabolic processes are also stimulated by mTORC1 action (32). Moreover, it was demonstrated that mTORC1 signaling is strongly implicated in the aging process of diverse organisms, including yeast, worms flies, and mammals (33).

On the other hand, mTORC2 is activated by growth factors but unlike mTORC1 its activity is not regulated by amino acids. mTORC2 phosphorylates PKC-α, AKT/PKB (Ser473) and paxillin (focal adhesion-associated adaptor protein) (Tyr118), to regulate the activity of the small GTPases Rac and Rho, controlling cell survival and cytoskeletal organization and cell migration (34).

## Regulation of mTORC1 Signaling in Cancer

The mTORC1 is often deregulated in numerous cancer types, such as breast, cervical cancer, esophageal squamous cell carcinoma, lung and hepatic cancers (35–39). mTORC1 is often activated by mutations in its upstream regulators. These include gain-of-function mutation of PI3K and loss-of-function mutation of the tumor suppressor PTEN (40). In a number of *in vitro* cell-lines and *in vivo* murine xenograft models, it has been demonstrated that aberrant mTORC1 contributes to tumor growth, angiogenesis, invasion and metastasis (41). Given its key role in the regulation of process associated with cell growth and metabolism in cancer, specifically with the mitochondrial functions, we focus on mTORC1.

It has been shown that mTORC1 is regulated by growth factors through the phosphoinositide 3-kinase/protein kinase B, also known as Akt (PI3K/PKB or Akt) pathway and by Ras/mitogen-activated protein kinase (MAPK) pathway (42). Binding of insulin or insulin-like growth factor (IGF) to their receptor lead to recruitment and phosphorylation of the insulin receptor substrate and subsequent recruitment of PI3K. PI3K phosphorylates the inositol ring of the membrane phospholipid, phosphatidylinositol-4,5-biphosphate (PI-4,5-P_2_) to generate phosphatidylinositol-3,4,5-trisphosphate (PIP_3_) at the cytoplasmic side of the cellular membrane (43). PIP_3_ recruits a subset of pleckstrin homology (PH) domain-containing proteins, such as the same protein kinase Akt and constitutively active phosphoinositide-dependent kinase 1 (PDK1) (44, 45). In turn PDK1 phosphorylates Akt in T308 (46), however the maximal activation of Akt requires its additional phosphorylation on S473 located at the carboxyl-terminus site, mediated by mTORC2 (47). Akt inhibits the tuberous sclerosis complex (TSC) that limit the mTORC1 signaling, TSC complex is composed by three subunits: TSC1 (Harmatin), TSC2 (Tuberin), and TBC1D7 (48, 49). Akt phosphorylate TSC2 on five residues (S939, S981, S1130, S1132, and T1462) leading to its inactivation (50, 51). The TSC complex is a negative regulator of the small GTPase Rheb (Ras homolog enriched in brain) (52), via stimulation of GTP hydrolysis. On the other hand Rheb-GTP is translocated to the lysosomal membrane, where directly interacts with the catalytic domain of mTOR promoting its activation (53). Once mTORC1 is activated, positively controls cell growth through stimulation of protein synthesis by induction the phosphorylation of its two main targets, the eukaryotic initiation factor 4E binding protein 1 (4E-BP1), and the ribosomal protein S6 kinase (S6K). Raptor-mTOR binds to S6K and 4E-BP1 through their respective TOR signaling (TOS) motifs (54, 55) enhancing translation of proteins involved in the control of cell growth/size and cell cycle progression.

The 4E-BPs are small (~15–20 kDa) proteins that interact with eukaryotic translation initiation factor 4E (eIF4E) inhibiting translation initiation, this being a very important regulation point in protein translation. Although there are three 4E-BPs known isoforms in mammals (4E-BP 1, 2, and 3), most studies had focus on 4E-BP1. mTORC1 phosphorylates 4E-BP1 in Thr37/Thr46, followed by Thr70, and finally Ser65 (56). Phosphorylation of Thr70 and Ser65 are part of the response to extracellular signals such as serum stimulation. Phosphorylation of all of these sites inhibits 4E-BPs' binding to eIF4E. The 4E-BPs prevents the formation of the translation initiation complex (eIF4F) by competing with eIF4G for binding to the dorsal side of eIF4E and reduces cap-dependent translation initiation (57). On the other hand the ribosomal protein S6 kinase (rpS6) known as S6K was first identified in unfertilized *Xenopus laevis* eggs as a 90 kDa polypeptide, termed p90 or rpS6 kinase (RSK, also known as p90RSK) (58). Later a protein with a molecular weight of 65–70 kDa was purified from chicken embryos and 3T3 cells, and referred to as S6K (59). Mammalian cells express both S6K1 and S6K2 also known as S6Kα and S6Kβ, respectively, which are encoded by two different genes and share a very high level of overall sequence homology (60). S6K1 has cytosolic and nuclear isoforms (p70 S6K1 and p85 S6K1, respectively) (61), whereas both S6K2 isoforms (p54 S6K2 and p56 S6K2) are primarily nuclear. S6K was identified as the main kinase responsible for ribosomal protein S6 phosphorylation (60), S6K regulates the mRNA biogenesis, translation initiation, and elongation.

## Mitochondria and Cancer

**Figure 2:**
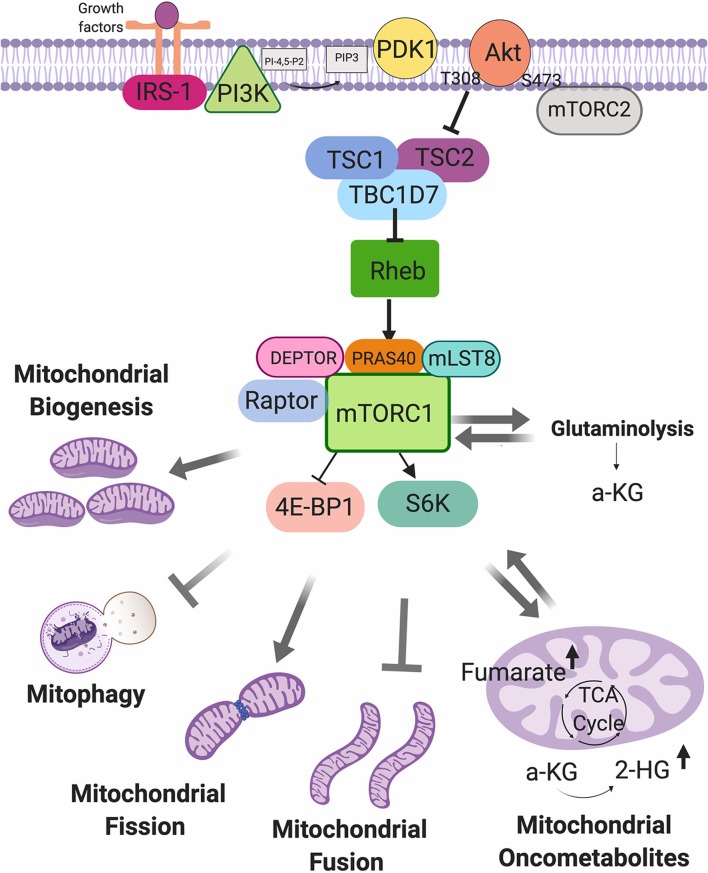
Mechanistic target of rapamycin complex 1 (mTORC1) as a regulator of mitochondrial functions. mTORC1 can be activated by growth factor, and can regulate the mitochondrial biogenesis, mitophagy, fission and fusion processes, glutaminolysis, and mitochondrial oncometabolites generation.

In addition to genetic aberrations, tumor cells rewiring their metabolism, such as aerobic glycolysis, glutamine uptake, accumulation of intermediates of glycolysis, and upregulation of lipid and amino acid synthesis, and OXPHOS, enable support their high demands on nutrients for building blocks and energy production (62). In cancer development tumor cells reprogram their metabolism to guarantee survival and proliferation in an often nutrient-scare and stressful microenvironment. (40). Moreover, several findings demonstrate that mutations in oncogenes and /or tumor suppressor genes can mediate metabolic rewiring in cancer cells to support the high demands for building blocks and energy production (63). Tumor cells acquire a metabolic plasticity that allows alternate between aerobic glycolysis and OXPHOS in order to maintain malignant phenotypes, such as a chemotherapy resistance, tumor invasion, and metastasis, and mitochondria play a central role in this dynamic (64).Changes in mitochondrial respiration rates are accompanied by changes in mitochondrial mass, the rate of fission, fusion, mitochondria biogenesis and mitophagy as well as mtDNA copy number, transcription and translation (64). In recent years, several evidences have established the role of mTORC1 as a central regulatory node in such events, which coordinates energy consumption by the translation apparatus and ATP production in mitochondria (65) (Figure 2).

It has been demonstrated that the role of mitochondria in cancer can vary depending of input genetic, environmental, and tissue-of-origin between tumors (4). The mitochondrion, contains its own DNA (mDNA) which is replicated independently of the host genome, mDNA comprises a circular genome of 16, 569 base pairs and encodes 37 genes, including 13 subunits of the electron transport chain (ETC), 2 ribosomal RNAs and 22 tRNAs, the remaining mitochondrial proteins are encoded by the nuclear genome and are imported into the mitochondria (66). Higher mtDNA copy number and mitochondrial function may confer an invasive advantage to human colorectal cancer (67).

Respiratory chain protein complexes (complexes I-IV) are placed into the inner membrane of mitochondria together with adenosine triphosphate (ATP) synthase, protein import machinery and transport proteins regulating metabolites passage through the matrix. The generation of ATP in mitochondria is coupled to the oxidation of NADH and FADH_2_, and reduction of oxygen to water (68). Abnormalities in mitochondrial complex I activity increase the aggressiveness of human breast cancer cells (69). The complex I, II and IV have all been shown to be hyperactive in human breast cancer cells; compared to tumor stromal cells and normal epithelial ductal cells (70). Interestingly it was shown that COX7RP is overexpressed in breast and endometrial cancer cells and promotes *in vitro* and *in vivo* growth by stabilizing mitochondrial supercomplex assembly even in hypoxic states, and increases hypoxia tolerance (71). Recently it was shown that OXPHOS is regulated by fascin, an actin-bundling protein that promotes lung cancer metastatic colonization by augmenting metabolic stress resistance by remodeling mitochondrial actin filaments (72).

The wide regulation of the mitochondria in cancer is of great importance and is a promising target in the development of cancer therapy (73), a number of therapeutic strategies have been based on targeting tumor mitochondrial proteins and their functions, such as metformin that has had currently a lot of impact on cancer therapy (74). Metformin induce the inhibition of OXPHOS due to reduced function of mitochondrial complex I underlies cellular and whole organism actions (75), this topic will be reviewed later in this review.

## mTORC1 and Mitochondrial Regulation by miRNAs in Cancer

The expression of a large number of oncogenes and tumor suppressor genes is regulated by miRNAs, which altered expression, is currently though as a hallmark of cancer. miRNAs or microRNAs are small non-coding RNAs (21–25 nt), that regulate gene expression by targeting mRNAs for degradation or suppressing translation (76). In cancer, miRNAs are divided into two categories, oncogenic miRNAs and tumor suppressor miRNAs, which are up regulated and down regulated during tumorigenesis (77). According to its role as a master regulator of cell growth, mTORC1 activity is modulated by different extracellular signals and intracellular mechanisms, interestingly it has been shown that some miRNAs can also regulate the mTORC1 activity directly or through targeting upstream regulators such as PI3K/Akt pathway. For instance, miR-451 is upregulated in glioma compared with control brain tissue; furthermore decreased miR-451 expression was associated to a suppressed tumor cell proliferation via CAB39/AMPK/mTOR pathway in two glioma cell lines (78). Furthermore, over expression of miR-405 promoted caspase-3/-9 and Bax protein expression, and suppressed cyclin D1 protein expression and the PI3K/Akt/mTOR pathway inhibiting cell proliferation and promoting cell apoptosis in gastric cancer-derived cells (78). On the other hand evidence shown that mTORC1 regulates miRNAs biogenesis and given the broad function of miRNAs in cancer development, it is possible that a significant portion of mTORC1 function, may be through its ability to control miRNA biogenesis. It was shown that chronic treatment with rapamycin leads to significant alterations in miRNA profiles and these changes correlate with resistance to rapamycin. The miRNAs associated to rapamycin resistance were miR-370, miR-17-92 and its related miR-106a-92, and miR-106b-25 clusters, which have been shown to have oncogenic properties in several types of cancer (79). Ye and collaborators (2015) report that mTORC1 activation downregulates miRNA biogenesis through upregulation of Mdm2, which is a necessary and sufficient E3 ligase for ubiquitinylation of Drosha an essential RNase dedicated to processing pri-miRNA in response to the cellular environment (80). On the other hand it was shown that mTORC1 in TSC2 deficient cells, promotes the miRNA biogenesis through of GSK3β regulation. mTORC1 induces the activity of the microprocessor, a nuclear complex that includes the nuclease Drosha and its partner DGCR8, this complex cleaves the stem loop of pri-miRNA to form premiRNA via the nuclease activity of Drosha (81).

On the other hand it was reported that several miRNAs targeting several mRNAs of nuclear-encoded mitochondrial proteins, integrating miRNAs into the landscape of translational regulation of mitochondrial functions such as TCA cycle, production of ROS and glutamine metabolism and mitochondrial fission process (82). miR-125a is frequently downregulated in several human cancer such as ovarian, non small-cell lung and gastric cancer and colorectal cancer (83–85) Moreover low expression of miR-125a is associated with increased tumor diameter, high Ki67 expression and poor overall survival of patients with gastric carcinoma (86) Additionally miR-125a deficiency enhances agiogenic processes through metabolic reprogramming of endothelial cells (87). Interestingly it was demonstrated that miR-125a is decreased in pancreatic cancer cells (PANC-1), accompanied by an increase in the contents of mitofusin 2 (MFN2) an important regulator of mitochondrial fission. Interestingly reintroduction of miR-125a triggered mitochondrial fission via downregulation of MFN2. Excessive mitochondrial fission contributes to activation of mitochondria-dependent apoptosis and impairs cellular migration via induction of F-actin degradation (88).

miRNAs are encoded in the nuclear genome and exported to the cytosol where they perform most of their functions, however, the expression of miRNAs within the mitochondrion has been recently demonstrated, which can be either mitochondrial encoded or transcribed within the nucleus and subsequently localized to mitochondria, this miRNAs are termed as mitomiRs (89). MitomiRs are likely to contribute to some post-transcriptional regulation of gene expression related to the mitochondrial functions (90). Interestingly mitomiRs have been shown to play a very important role in chemotherapy resistance through the regulation of metabolic changes. For instance, it was demonstrated that mito-miR-2392 regulates the cisplatin resistance by reprogramming the balance between OXPHOS and glycolysis in tongue squamous cell carcinoma (TSCC) cells. Furthermore, in a retrospective analysis of TSCC patient tumor revealed a significant association of miR2392 and the expression of mitochondrial gene with chemosensitivity and overall survival (91).

Although several cancer processes are regulated by miRNAs, there is a lacking of investigation aimed to determine the role of the mitomiRs and mTORC1 regulation either, in metabolic responses to therapy as well as mitochondrial functions, representing an open opportunity for future research.

## mTORC1 Regulates Translation of Mitochondrial Proteins Encoded in the Nuclei

Protein synthesis or mRNA translation, is the major energy-consuming process in the cell (92, 93). It is well-established that deregulation of mRNA translation is a prominent characteristic of cancer cells (94). Protein translation can be simplified into four stages: initiation, elongation, termination, and ribosomes recycling, however the critical regulation point occurs in the step of mRNA translation initiation, this step is regulated by several key signaling pathways, including PI3K/Akt/mTORC1 that in fact are over expressed in several neoplasms (95). The mitochondrial translation comprises the same four stages, although mitochondria have their own translation machinery with distinct mitochondrial ribosomes (mitoribosome), tRNAs and translation factors than the cytosolic counterparts. Yet the majority of the mitochondrial proteins, including all factors required for mtDNA maintenance and expression, and some components of the ETC complexes are encoded in the nuclear genome (96) and are translated in cytosolic ribosomes, and transported into mitochondria via peptides that function as import signals, this mitochondrial proteins are widely regulated via mTORC1 (97). Since mTORC1 regulates the cellular most energy consuming process, it is reasonable that mTORC1 responds to bioenergetics variation, a process controlled by mitochondria. Additionally, it was shown that mTORC1 regulates the capacity of the mitochondria to produce ATP as well as cell cycle progression in cancer cells (98).

Larsson et al. (99) evaluated the impact of different mTORC1 inhibitors in the global regulation of protein translation in MCF7 cells, interestingly, the authors found several mRNAs involved in mitochondrial functions (99). In another study, it was demonstrated that mTORC1 regulates the translation of the ATP synthase components, included ATP synthase subunit delta (ATP5D), and the transcription factor A, mitochondrial (TFAM), which promotes mitochondrial DNA replication and transcription through 4E-BPs, moreover, this was related with a higher mitochondrial activity (100). In conclusion, there is a feed-forward mechanism in the cells whereby translation of nucleus-encoded mitochondria-related mRNAs is regulated via mTORC1/4E-BP pathway to induce mitochondrial ATP production capacity and thus provide sufficient energy for protein synthesis (100). In support with this, using nano-cap analysis, which allows determination of transcription start sites on a genome-wide scale, a large number of non-TOP mRNAs were found to be mTOR sensitive (101). Among these non-TOP mRNAs, mRNAs with short 5′ UTRs were in fact mRNAs encoding for protein involved in mitochondrial functions, including components of the respiratory chain complexes (ATP50, ATP5D, UQCC2) (101).

This demonstrates that mTORC1 drives cell proliferation and neoplastic growth not only by inducing the translation of genes involved in cell growth but also by promoting the translation of mitochondrial proteins involved in cellular energy production, proteins implicated in mitochondrial DNA replication and mitochondrial repair, transcription, and translation.

## Mitochondrial Localization of mTORC1: Regulation of the Mitochondrial Oxidative Metabolism

As described previously, mTORC1 regulates the translation of mitochondrial proteins encoded in the nucleus, however it is not the only function by which this important metabolic regulator acts. Interestingly, it has been shown that mTORC1 is found in mitochondrial fractions suggesting a regulatory ATP producing capacity.

Desai et al. (102) described the first association between mTOR and mitochondria through subcellular fractionation of human T cells. They identified that mTOR co-interact with purified mitochondria elements, and specifically mTOR is associated with the outer mitochondrial membrane. In addition, they demonstrated that when treating with mitochondrial inhibitors, the activity of mTORC1 was decreased (102). In support of these data, another study showed that mTOR-raptor complex is also present in the mitochondrial fraction of Jurkat T cells; this complex was tightly correlated with mitochondrial activity, specifically with high consumption of oxygen and mitochondrial membrane potential as well as with a higher capacity for ATP production. Moreover, disruption of the mTOR-raptor complexes with rapamycin or with RNAi resulted in a decreased mitochondrial metabolism (103).

The voltage-dependent anion channels (VDACs) are pore forming proteins found in the outer mitochondrial membrane of all eukaryotes, and are the binding sites for several cytosolic enzymes, including the isoforms of hexokinase and glycerol kinase, allowing a preferential access to mitochondrial ATP (104). This mitochondrial protein is often overexpressed in several cancers, and it has been shown that VDAC1 depletion leads to a rewiring of cancer cell metabolism in breast cancer, lung cancer and glioblastoma, resulting in cell growth arrest, and tumor growth inhibition (105). Ramanathan et al. (106) showed that leukemic cells treated with rapamycin, showed a decreased mitochondrial activity. Interestingly, they found that mTOR coimmunoprecipitates with the VDAC1 and with the anti-apoptotic protein B-cell lymphoma-extra-large (Bcl-xl). They also demonstrated that mTOR phosphorylates Bcl-xl in serine 62 and increases its activity. Since Bcl-xl is a key mediator of mitochondrial function and cellular apoptosis that has been shown to bind to VDAC1 and increase the substrate permeability, its suggested that mTOR could control mitochondrial metabolism in a Bcl-xl-VDAC1 dependent manner (106). On the other hand, it was demonstrated that under radiation stress, mTOR relocates to mitochondria in MCF7, HCT116, and U87 cells, where it interacts with hexokinase II, an enzyme that phosphorylates glucose during glycolysis switching bioenergetics from aerobic glycolysis to OXPHOS which is related to an increased tumor resistance to radiation treatment (107), this interaction was also observed in another study in neonatal rat ventricular myocytes under glucose starvation (108). In another study, it was demonstrated that mTOR/Akt pathway regulates the mitochondrial respiratory activities and the expression of complex I, II and IV of the electron transport chain trough 4E-BP1 (109). Furthermore, another study suggested that mTOR-raptor may acts as a metabolic checkpoint in G1 phase of cell cycle by regulating mitochondrial function (110).

Triple-negative breast cancer cells possess special metabolic characteristics compared to estrogen receptor (ER) positive cells, manifested by high glucose uptake, increased lactate production, and low mitochondrial respiration which is correlated with attenuation of mTOR pathway and decreased expression of p70S6K. Re-expression of p70S6K reverses their glycolytic phenotype to OXPHOS state, while knockdown of p70S6K in ER positive cells leads to suppression of mitochondrial OXPHOS (111). It was demonstrated that global targeting of mTOR caused both anti-survival and pro-survival mitochondrial response, which were differentially exhibited in diverse cancer cells according to their intrinsic sensitivity to mTOR inhibition and hyperactive PI3K/AKT/mTOR activity status and/or growth factor-dependence (112).

## mTORC1 and Mitochondrial Dynamic in Cancer

The mitochondrial dynamic is a balance between fission and fusion processes (113). Mitochondria fusion is the union of two mitochondria resulting in one mitochondrion; organelle movement along cellular tracks that permit the encounter between two different mitochondria facilitating the fusion process (114). Fusion helps cells to mitigate stress by sharing multiple elements, which sustain mitochondrial biology as a form of complementation. Mitochondrial fusion involves two sequential steps: first, the outer membranes (OMs) of two mitochondria fuse; second, the inner membranes (IMs) fuse. OM fusion is mediated by mitofusin 1 (MFN1) (115) and MNF2 (116), which are dynamin-related GTPases at the OM (117). IM fusion is mediated by the dynamin-related protein optic atrophy 1 (OPA1) (118).

On the other hand, the mitochondrial fission is characterized by the division of one mitochondrion in two daughters, this process is required for segregation of damaged mitochondria for mitophagy, mtDNA replication, and mitochondria redistribution and motility during cell division (113). The fusion process requires the recruitment of dynamin-related protein 1 (DRP1) (119) from the cytosol to the mitochondrial OM. Assembly of DRP1 on the mitochondrial surface causes constraint of the mitochondria and leads to division of the organelle (120). In mammals exist four DRP1 receptors: mitochondrial fission 1 (FIS1) (121), mitochondrial fission factor (MFF) (122), Mitochondrial dynamics proteins of 49 kDa (MID49), and MID51 that are located on the mitochondrial OM (123).

It has been established that the alteration of mitochondrial dynamics impact tumor development broadly. Alterations to the mitochondrial dynamic network also result in specific therapeutic susceptibilities, in particular, tumors with increased mitochondrial fragmentation or connectivity are hypersensitive to SMAC mimetics and induce apoptosis by blocking the action of inhibitor of apoptosis proteins (IAPs) (124). On the other hand, it was demonstrated that Drp1 expression was strongly increased in distant metastasis of hepatocellular carcinoma (HCC) compared to primary tumors. In contrast, Mfn1 showed an opposite trend (125). Moreover, *in vitro* experiments with HCC cells, demonstrated that mitochondrial fission significantly promoted the reprogramming of focal adhesion dynamics and lamellipodia formation mainly, by activating the CA^2+/^CaMKII/ERK/FAK pathway, which was associated with a greater capacity for migration and invasion (123, 125).

A very important protein in mitochondrial fission is the mitochondrial fission process protein 1 (MTFP1), also called mitochondrial fission process 1,18 kDa (MTFP18), an integral pro-fission protein located at the mitochondrial inner membrane whose loss results in a hyperfused mitochondrial reticulum, whereas its overexpression produces mitochondrial fragmentation (126). As mentioned earlier, mTORC1 promotes the translation of mitochondrial proteins encoded in nuclei, interestingly, using a genome-wide polysome profiling and translatome, it was demonstrated the treatment with rapamycin, PP242 and metformin (mTORC1 inhibitors) suppressed the translation of MTFP1 (99). Morita and collaborators recently demonstrated that mTORC1 is a regulator of mitochondrial dynamics and cell survival via MTFP1 translation. Using mouse embryonic fibroblasts (MEFs) and human malignant melanoma cells treated with active-site mTOR inhibitor (asTORi), was demonstrate that mTORC1 stimulates the translation of MTFP1 mediated by 4E-BP, and therefore the mTOR inhibition induces the phosphorylation of the DRP1 at Ser 637, this phosphorylation prevents it translocation to mitochondria, conversely, the pro-fission phosphorylation site of DRP1 at Ser 616 was decreased in asTORi treated cells. This process was associated with a high mitochondrial elongation, branching, and circularization (127).

In support with these results it has been shown that cellular starvation inhibits mTORC1 pathway, interestingly, it was shown that the cells show a mitochondrial elongation phenotype under starvation (128, 129) similar to that observed in asTORi treatment. Combination between mTOR inhibitors and an increase of mitochondrial fission activates cell apoptosis, converting the mTOR inhibitors action of cytostatic to cytotoxic (127). In other study, it was shown that S6K1 contributes to mitochondrial dynamics, homeostasis and function, since MEFs-lacking S6K1 exhibited more fragmented mitochondria and a higher level of Drp1 with greater phosphorylation levels in Ser 616 (130). The depletion of S6K1 induced mitochondrial fission but not mitophagy. These changes in mitochondrial morphology alter its function disrupting the balance of OXPHOS, ATP production and changing cellular energy metabolism (130).

## Mitochondrial Biogenesis and Mitophagy: mTORC1 in Cancer

Mitochondrial mass is regulated by two opposite pathways, mitochondrial biogenesis and mitophagy, both processes emerging as dual regulators of tumorigenesis (4). Mitochondrial biogenesis is the growth and division of pre-existing mitochondria, whereas mitophagy is a form of autophagy that selectively degrades damaged mitochondria (131).

Mitochondrial biogenesis is widely regulated at transcriptional, translational and post-translational levels. Peroxisome proliferator-activated receptor γ co-activator 1α (PGC1-α) and related transcription co-activator are the master transcriptional regulators of mitochondrial biogenesis (132). PCG1-α binds to various transcription factors and nuclear receptors that recognize specific sequences in their target genes and promotes the mitochondrial biogenesis and oxidative phosphorylation in cancer cells and also promotes tumor metastasis (133) and drug resistance in colorectal cancer cells by regulating endoplasmic reticulum stress (134). The targets of PGC1-α include enzymes of energy metabolism as well as essential factors for the replication and transcription of mtDNA. PGC1-α is a transcription factor for mitochondrial genes, which action depends on its association with other transcription factors such as yin-yang (YY1), nuclear respiratory factor 1(NRF1) and 2 (NRF2), estrogen-related receptor α (ERRα) (132, 135). YY1 is a zinc finger protein and a member of the GLI-Kruppel family that can activate or inactivate gene expression depending on its interacting partners (136), YY1 is overexpressed in multiple cancer types and correlates with poor clinical outcomes (137, 138). However, other papers report that YY1 inhibits the cell growth in different tumor cell types *in vitro*, including human breast carcinoma cells and glioblastoma cells (139).

Using skeletal muscle cells was showed that rapamycin decreased the expression of the PGC1-α, RREα, and NRF1 in correlation with decreased oxygen consumption. Moreover, it was identified that mTOR-raptor complex interacts with YY1, and in association with PCG1-α, regulates the mitochondrial gene expression (ATP5G1, Cox5A, cytochrome c, NDUF88, and UCP2) (140). In support with this results, it was demonstrated that mTOR induces the phosphorylation of YY1 (T30 and S356) consequently favoring the interaction with PGC1-α and increased mitochondrial morphology and bioenergetics state, in skeletal muscle (141). These results demonstrate that mTORC1 regulates mitochondrial biogenesis by promoting the transcription of mitochondrial genes. On the other hand, mTORC1 controls mitochondrial activity and biogenesis by selectively promoting translation of nucleus-encoded mitochondria related mRNAs via inhibition 4E-BPs. Moreover, the stimulation of the translation increases ATP production capacity, a required energy source for translation in MCF7 cells (100). In addition to stimulation of mitochondrial biogenesis by antagonizing 4E-BP1 dependent translation repression of mitochondria related mRNAs, mTORC1 inhibits mitochondrial degradation by suppressing autophagy (100).

PGC-1β is also an important mitochondrial biogenesis regulator, through regulation of the expression of NRF1 (142). It was shown that the levels of PGC-1β and mTOR correlated with overall mitochondrial activity in breast cancer samples. Moreover, the knockdown of endogenous PGC-1β, leads to a decreased expression of mTOR pathway related genes and induces apoptosis in MDA-MB-231 cells (143). Interestingly, it was demonstrated that the branched chain amino acid transaminase 1 (BCAT1) actives mTORC1 and in consequence promotes the mitochondrial biogenesis, ATP production and defense of oxidative stress (143). The inhibition of mTORC1 with rapamycin, neutralized the roles of BCAT1 in mitochondrial function and breast cancer cell growth (143). Recently, it was shown that rapamycin, enhanced the processes of apoptosis and initiation of autophagy in LKB1 deficient urothelial carcinoma of the bladder both *in vitro* and *in vivo*, which was associated with deregulated mitochondrial biogenesis and AMPK activation (144). These results are relevant because AMPK is an important regulator of mitochondrial biogenesis via PGC1-α (145), which also inhibits the mTORC1 pathway.

Mitophagic status was assessed in a panel of human cytoplasmic hybrid (cybrid) cell lines carrying a variety of pathogenic mtDNA mutations. It was found that both genetic and chemically induced loss of mitochondrial transmembrane potentially caused recruitment of the pro-mitophagic factor Parkin to mitochondria but it was insufficient to prompt mitophagy. They found that mitophagy could be induced following treatment with the mTORC1 inhibitor rapamycin (146).

**Figure 3:**
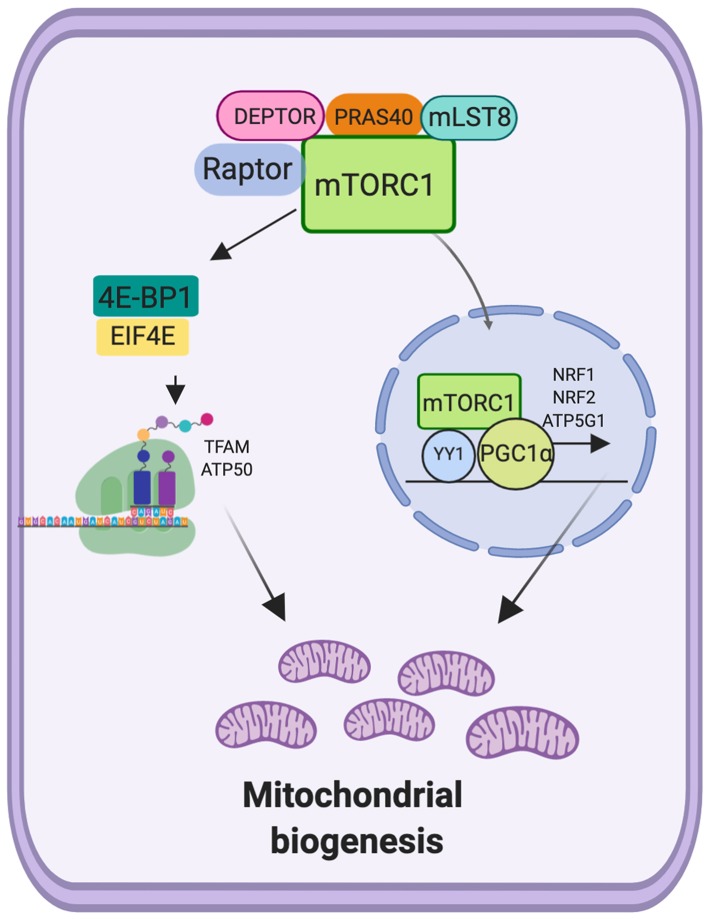
Mechanistic target of rapamycin complex 1 (mTORC1) and mitochondrial biogenesis. mTORC1 promotes mitochondrial biogenesis via upregulation of translation genes and moreover via transcriptional regulation of TFAM, ATP50, NRF1, NRF2 genes.

These findings suggest that, mTORC1 is an important regulator of mitochondrial biogenesis, by regulating the expression of important factors in the regulation of mitochondrial biogenesis, both at the transcriptional level and at the translation level (Figure 3).

## Glutaminolysis and mTORC1 in Cancer

Glutaminolysis is a set of reactions that occurs in mitochondrial matrix and cytosol in proliferating cells. In such reactions, the amino acid glutamine is degraded to glutamate, ammonium, aspartate and pyruvate, among others. Glutamine, glutamate as well as aspartate, are used for nucleic acid synthesis, other important function of glutamine is replenishing the TCA cycle intermediate α-KG.

It has been reported that glutamine is the amino acid most frequently found in plasma and muscle (147), glutamine concentration ranges from 450 to 800 μM in human plasma (148). Glutamine has been defined as a non-essential amino acid; nevertheless, evidence has showed that glutamine becomes essential in stressful conditions (149). As an example, when cells are under hypoxic stress, glutamine-derived α-KG is used to stimulate lipids synthesis (150). Carbon and nitrogen from the glutamine present in blood are used for biosynthesis and also for providing energy to the cell (151). Specifically, glutamine is the leading donor of nitrogen for purine and pyrimidine nucleotide synthesis, as well as a supplier for amino groups for non-essential amino acids synthesis, such as aspartate, alanine, glycine and serine, moreover, nitrogen from glutamine participates in nucleic acid and *de novo* protein synthesis (149, 152). Finally, the glutamine-derived carbon is source for fatty acid and amino acid synthesis as well (151).

Glutamine enters to the cells via SLC (solute carrier)-type transporters. Fourteen of these transporters are known for transporting glutamine to the plasma membrane which are classified into four families: SLC1, SLC6, SLC7, and SLC38 (153). Glutamine is metabolized within the mitochondrion via two deamination steps. The first one produces glutamate through an irreversible reaction catalyzed by glutaminase (GLS1 and GLS2 in mammals); in the following deamination reaction, α-KG is produced by the enzyme glutamate dehydrogenase (GDH) (154). The α-KG generated by glutaminolysis is a major anaplerotic source in the TCA cycle.

Importantly, it was demonstrated that glutamine could be useful for cancer cells to drive tumor growth due to is used for energy generation as well as for biomass accumulation being a source of carbon and nitrogen as mentioned before (152), moreover glutamine can be consumed by proliferating cells more rapidly than needed to satisfy nitrogen requirements (155). As a result of glutamine depletion, most cancer patient's loss body weight due to muscle mass consumption provoking weakness, all these symptoms are known as cachexia (155, 156). It is important to notice that, when cancer cells are deprived of glutamine, undergo cell cycle arrest due to nitrogen deficiency since nitrogen is necessary for nucleotides synthesis (157). In 1978, Lawrence et al. observed that glutamine is the major energy source in HeLa cell line (158). Additionally, evidence supports that glutaminolysis provides metabolites, such as glutamate to promote tumor growth, as observed by Dornier et al. (159). The group investigated the participation of glutamine metabolism in invasive processes so that, they showed that mammary epithelial cells from normal tissue uptake glutamine, yet glutamate secretion was not observed. Extracellular glutamate is needed at low concentrations for mammary epithelial phenotype maintenance, but higher concentrations promote key characteristics of the invasive phenotype, moreover, in primary cultures of invasive breast cancer cells it was observed a high conversion glutamine to glutamate (159).

**Figure 4:**
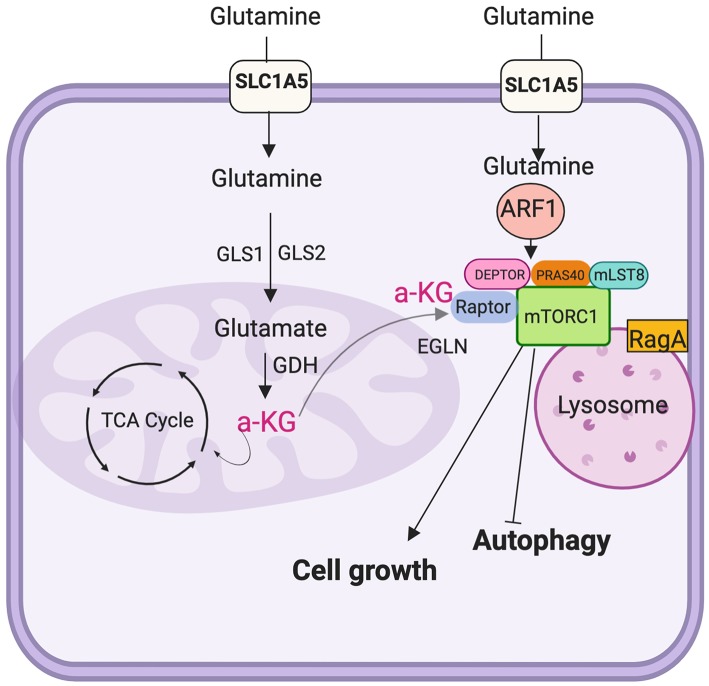
Glutaminolysis and Mechanistic target of rapamycin complex 1 (mTORC1) The α-ketoglutarate (α-KG) produced by glutaminolysis is used for tricarboxilic acid (TCA) cycle intermediates replenish, a process known as anaplerosis. Once α-KG is exported from the mitochondria to the cytosol activates EGLNs, which in turn triggers mTORC1 activity promoting cell growth and inhibits autophagy.

Autophagy and cell growth are found to be under control of mTORC1; those two cellular processes are regulated by glutaminolysis, so that mTOR activity is tightly controlled to prevent inappropriate cell growth (Figure 4). In fact, it has been found an upregulation of mTORC1 in several cancers and such activation is required for cell growth and protein synthesis. Further, glutamine metabolism is found disrupted in several cancer types, including papillary thyroid cancer where using cell lines was demonstrated that such cells are dependent on glutamine and glutaminolysis-associated proteins.

Through different experimental approaches, an aberrant overexpression of GLS was showed in cancer; moreover, pharmacological inhibition (by using inhibitors BPTES and CB-8939 that target both isoforms of GLS) and genetic knockdown of GLS repressed glutaminolysis and diminished mitochondrial respiration. Additionally, using tissues and cells from patients with papillary thyroid cancer, an altered overexpression of glutaminase was observed. When GLS was inhibited using a siRNA, mTORC1-signaling pathway was deactivated leading to an increase of autophagy and apoptosis (160).

It has been demonstrated that arginine and leucine prompt mTORC1 by activating RAS-related GTPase (RAG) complex; as a result, mTORC1 is recruited and triggers lysosome activity. Studies have demonstrated that glutamine positively regulates the mTORC1 pathway when promoting leucine uptake (161) and as well-boosting mTORC1 assembly as well as its localization into the lysosome; indeed, the presence of α-KG is considered to be enough to promote mTORC1 localization into the lysosome (162) (Figure 3).

The mentioned RAG-dependent regulation of mTOR could rely on glutamine, arginine and leucine transporter SLC38A9 (163–165). Although the mechanism is not well-understood, it has been hypothesized that α-KG could be able to regulate RAGB activity as well as mTOR activation at a downstream glutamine metabolism level (151). On the other hand, Jewell and her group reported in 2015 that mTORC1 activation could be independent of the Rag GTPases and supported the fact that mTORC1 is differentially regulated by the amino acids leucine and glutamine. Using mouse embryonic fibroblasts RagA and RagB knockout cells, they demonstrated that leucine stimulates mTORC1 by Rag GTPase-dependent mechanism meanwhile glutamine stimulates mTORC1 through a mechanism that is carried out by Rag in an GTPase-independent mechanism in order to translocate mTORC1 to the lysosome (166).

In 2013, Csibi and collaborators reported that mTORC1 pathway regulates glutamine uptake and metabolism. The results showed that the mTORC1 pathway negatively controls SIRT4, an ADP-ribosyltransferase that is found in the mitochondria and inhibits glutamine dehydrogenase (GDH), through stimulation of proteasome-mediated degradation of cAMP-responsive element-binding (CREB) 2. In fact, it has also been reported that SIRT4 expression is decreased in several human cancers (167).

The same research group postulated a previous model in which they concluded that cells are addicted to glutamine as a result of mTORC1 activation (168). It was shown that α-KG could be exported to the cytosol by the mitochondrial carrier protein α-KG/malate named SLC25A11(154). At high glutaminolyc rate, cytosolic α-KG activates the enzymes that function as oxygen and nutrient of the cell sensors EGLNs (prolyl hydroxylase enzymes PHD) such enzymes are required for mTORC1 activation-dependent of amino acids in a HIF-1α independent manner to promote cell growth and anabolism and inhibit autophagy (154, 162).

An elevated glutaminolysis is related to the promotion of cancer progression at early stages by stimulating cell growth through the mTORC1 pathway and diminishing elimination of altered proteins and organelles by inhibiting autophagy (154, 162). In another case, glutamine dependence was evaluated in six different cell lines from squamous cell carcinoma and it was found that five out of six cell lines were glutamine-dependent, also, glutamine depletion, using GLS1- inhibitors BPTES and compound 968, decreased cell proliferation in those five cell lines, meanwhile inhibition of cell proliferation in QG56 glutamine-independent cell line was not reported as significant. Further, it was observed that the inhibition of glutaminolysis suppressed mTORC1 activity, by evaluating pS6 levels in the glutamine-dependent RERF-LC-AI cell line but the activity of mTORC1 was not affected in the QG56 glutamine-independent cell line. Finally, inhibition of glutaminolysis induced autophagy in RERF-LC-AI cell line (169). Furthermore, the activation of mTORC1 inhibits the family of enzymes that catalyze phosphorylation of phosphatidyl inositol, one of the main phospholipids present in the cell, specifically at the d-3 position of the inositol ring, to generate PtdIns (3)P complex I and unc-51 like autophagy activating kinase complex (ULK), both proteins participate in the initiation step of autophagy and mTORC1 activation limits the initiation steps of autophagy. On the other hand, glutaminolysis products GSH, NADPH, and α-KG limit ROS production to prevent autophagy induction (154).

It has been observed that a reactivation of mTORC1 by glutaminolysis is also required for lysosome regeneration and autophagy termination (154). In the specific case of autophagy, it has been reported that autophagy has a dual role in cancer, acting as tumor suppressor in some cases. For instance, metabolic stress causes the expression of p62, a sustained autophagy substrate protein, resulting in autophagy defects and an altered expression of NF-kB, finally promoting tumorigenesis, this information indicates that autophagy suppresses tumorigenesis by limiting p62 accumulation (151, 170). On the contrary, autophagy seems to support cancer cells survival by facilitating nutrients and suppressing stress pathways. For instance, expression of H-ras and K-ras oncogenes in immortal non-tumorigenic baby mouse kidney epithelial cells upregulated basal autophagy promoting tumor cell survival (151, 171). Another interesting relation between mTOR pathway and autophagy is the association to lifespan and aging; mainly because it has been observed that inhibition of mTOR could bring as a consequence delay of aging due to autophagy stimulation resulting in a mitophagy increase (172). In fact, it is well-documented that inhibition of key components of mTOR and its counterpart in invertebrates TOR pathways, results in an extension of life span in part by the influence of mTOR on the called “hallmarks of aging,” an interesting an extensive review about this subject is broadly reviewed in Papadopoli publication (173).

The regulation of both, mTORC1 and glutaminolysis suggests that mTORC1 and glutaminolysis act in both directions hence they are found to regulate each other for promoting cell growth and cancer progression; mTORC1 also induces glutaminolysis by activating c-MYC-GLS and because c-MYC is GLS and GLUD1 transcription factor, glutamine metabolism is favored; additionally, the glutaminolysis-mediated activation of mTORC1 participates in autophagy inhibition and the DNA double-strand breaks sensor serine/threonine protein kinase ATM which participates in cell cycle delay after DNA damage. The mTORC1 pathway suppresses ATM via S6K1/2 signaling pathways and by upregulating mircroRNA-18a and microRNA-421 that target ATM (154, 174). An increase in glutamine synthetase abolishes the production of α-KG from glutaminolysis, as a result, an inhibition of mTORC1 is observed as well as an enhancement of autophagy, which is imperative for cancer cell survival (154, 175). There is an increasing interest in inhibiting simultaneously both, glutaminolysis and autophagy in order to trigger a synergistic effect that may be useful for patient outcome improvement and also to diminish toxicity.

A very interesting publication of 2016 shows that autophagy could be a survival mechanism upon rapamycin treatment. Interestingly, in conditions of nutrient restrictions, mTORC1 is activated by glutaminolysis during nutritional restrictions and autophagy is inhibited, so then apoptosis is induced, via upregulation of p62 in U-2 OS cells (176).

## Mitochondrial Oncometabolites and mTORC1

Dominant mutations in mitochondrial enzymes led to identification of mitochondrial derived signaling molecules, called oncometabolites. The term of oncometabolites refers to intermediates of metabolism that abnormally accumulate in cancer cells upstream or downstream of metabolic defects, often through loss-of-function or gain-of function mutations, respectively, in genes encoding the corresponding enzymes (177). This oncometabolites are 2-hydroxyglutarate (2HG), succinate and fumarate which have been demonstrated to contribute to the development and progression of cancer (178). The oncometabolites are produced by mutations in the nuclear-encoded TCA enzymes, isocitrate dehydrogenase 1 and 2 (IDH1/2), succinate dehydrogenase (SDH), and fumarate hydratase (FH) (177). Chin and co-workers discovered that metabolite α-KG increases the lifespan of adult *C. elegans* by inhibiting the highly conserved ATP synthase and mTORC1, mimicking dietary restriction in longevity (179). Interestingly, it has been shown that mTORC1 promotes the generation of oncometabolites in addition it was also shown that these oncometabolites regulate mTORC1, as a feedback regulation.

### 2HG and mTORC1

Isocitrate dehydrogenases 1 and 2 (IDH1, and IDH2) are key TCA cycle enzymes that are nicotinamide adenine dinucleotide phosphate (NADP^+^) dependent. IDH1 and 2 catalyze the oxidative decarboxylation of isocitrate to α-KG with production of reduced nicotinamide adenine dinucleotide phosphate (NADPH) (180). Mutations in IDH1 and IDH2 genes are mostly missense variants leading to a single amino-acid substitution of arginine residues at codon 132 in exon 4 of the IDH gene or codons 140 or 172 of the IDH2 gene. Mutant of IDH1 and IDH2 enzymes have a gain the function of catalyzing the reduction of α-KG to its (R)-enantiomer of 2-hydroxyglutarate (2HG), which accumulates to exceedingly high levels in patients with glioma, acute myeloid leukemia, esophageal squamous cell carcinoma (180–183) thus, 2HG levels being used as a biomarker for IDH mutation in these cancers (184). 2HG is an oncometabolite impairing epigenetic and hypoxic regulation through its binding to α-KG-dependent dioxygenases.

Recently, it was shown that 2HG induces angiogenic activity, because it induces the levels of secreted vascular endothelial growth factor (VEGF) in breast cancer cells, and finally enhance the endothelial cell proliferation and migration cell inducing MMP2 activity (185).

It was shown that both (R)-2HG and (S)-2HG bind and inhibit ATP synthase and mTOR signaling. Consistently, this inhibition is sufficient for growth arrest and tumor cell killing under conditions of glucose limitation in glioblastoma cells (186). Contrary to these results, it was demonstrated that mutations IDH1^R132H^ or IDH2^R172K^ in MEF and HeLa cells induce an increase in 2HG levels that stimulate both mTORC1 and mTORC2 signaling as highlighted by enhanced phosphorylation of p70S6K, pS6 and Rictor, and Akt, respectively. They also showed that 2HG inhibits the α-KG-dependent enzyme KDM4A and consequently, this affects the stability of DEPTOR a negative regulator of mTORC1 and mTORC2, leading to mTOR activation independently of the PI3K/Akt/TSC1-2 pathway (187).

In other study it was shown that rapamycin reduced 2-HG levels derived of lactate, in IDH1 mutant fibrosarcoma cell line (HT-1080 cells). Furthermore, they shown that rapamycin inhibit the growth in HT-1080 xenografts *in vivo* and 2HG production (188). In support with this, using two mutant cell lines for IDH and orthotopic mutant IDH tumor model, showed that the treatment with dual PI3K/mTOR inhibitor (XL765), induced a significant reduction in 2HG levels, and enhanced the survival (189).

### Fumarate and mTORC1

In the TCA cycle the reversible hydration of fumarate to malate is catalyzed by FH. The oncogenic properties of FH loss have been mostly associated with a high intracellular accumulation of fumarate. This oncometabolite shares structural similarity with another TCA cycle intermediate α-ketoglutarate, also referred to as 2-oxoglutarate (2-OG). 2-OG is a cofactor for a family of enzymes called 2-OG-dependent dioxygenases that catalyze the hydroxylation of a wide range of targets (190). The enzymes that belong to this family are the prolyl hydroxylases and the Jumonji C containing family of histone lysine demethylases and TET (ten-eleven translocases) enzymes (190). It was shown that high levels of fumarate inhibit the HIF-1α prolyl hydroxylases, leading to HIF-1α stabilization (191). HIF-1α is inactivated in normoxia by prolyl hydroxylase enzymes (PHD 1-3) using oxygen as a substrate. HIF-1α hydroxilated is associated to E3 ubiquitin ligase Von Hippel Lindau protein (VHLp) for its degradation, whereas in hypoxia condition stabilization and nuclear translocation occur, leading to oncogenes activation (192). HIF-1α is a transcription factor for metabolic genes such as hexokinase (HK), lactate deshydrogenase (LDHA) and glucose transporter (GLUT1) promoting tumor glycolysis (193). In other study it was demonstrated that fumarate accumulation promotes HIF-1α mRNA and protein accumulation independent of the VHL pathway but through an NF-kB dependent mechanism. Fumarate promotes p65 phosphorylation and p65 accumulation at the HIF-1α promoter through non-canonical signaling via the upstream Tank biding kinase (TBK1) promoting cell invasion of renal cancer cells (194). In accordance with the role of the fumarate accumulation with cytotoxicity and oncogenic capacity, it was demonstrated that cells exposed to high levels of fumarate and succinate lead to extensive DNA fragmentation and altering the global DNA methylation patterns via DNA hypermethylation in human hepatocellular carcinoma (195).

Interestingly it was shown that mTORC1 upregulation leads to accumulation of fumarate, and contributes to tumor transformation. Using a mouse model harboring the kidney specific inactivation of TSC1 that developed progressive renal lesions that eventually resulted in cortical renal papillary carcinoma, it was shown that TSC1 inactivation results in the accumulation of fumarate due to mTOR-dependent downregulation of the FH. The re-expression of FH rescued renal epithelial transformation (196). In support with these results, using primary samples from clear cell renal cell carcinoma (ccRCC) a total of 15 of 23 cancer samples displayed increased positive staining for pS6 protein (~65%), confirming mTORC1 upregulation in a large proportion of ccRCC cases. Among the 23 samples analyzed, 16 samples showed downregulation of FH mRNA levels compared with relative healthy tissue (196).

## mTORC1 as a Therapeutic Target

Chemotherapy and radiotherapy represent the leading option for cancer treatment and although responses are observed, relapses in several cancer types are common so then, effective therapeutic options for recurrent disease are lacking. There is a link among mTORC1 signaling upregulation and tumor growth, which establish that tumors could be responsive to mTORC1 inhibitors. The correlation between tumor growth and hyperactive mTORC1 signaling suggests that tumors may be sensitive to mTORC1 inhibitors. mTOR inhibitors are known primarily as cytostatic, so inhibiting cell growth could induce cell death when mTOR inhibitors are administrated alone or combined with different therapeutic drugs. Such inhibitors are a promising therapeutic strategy for treating several cancer types (197).

Rapamycin is the first known allosteric mTORC1 inhibitor studied, however, its poor water solubility and chemical stability have led to implement instead the use of semi-synthetic rapamycin analogs (or rapalogs) that show improved pharmacokinetic properties, solubility and reduced immunosuppressive effects (159, 160). To date, three rapalogs are being tested in clinical trials, CCI-779 (temsirolimus), AP23573 or MK-8669 (ridaforolimus), and RAD001 (everolimus) (198). Temsirolimus is an ester derivative drug, approved for renal-cell carcinoma patients since 2007, and is administrated to patients via intravenous or orally. Ridaforolimus was designed to improve aqueous solubility and is administered orally. And finally, everolimus is a hydroxyethyl ether derivative that is administrated to patients via oral (199). In addition, the prototype rapamycin (sirolimus) is also being tested in kidney transplant recipients, for preventing the occurrence of secondary skin cancers, which are common in these patients (200).

These drugs induce apoptosis inhibition by forming a complex with the intracellular immunophilin FKBP12 thus inhibiting the phosphorylation of the mTOR targets, S6K1 and 4E-BP1, as a result, the activation of cyclin-dependent kinases (CDK) is prevented, specifically, the expression of cyclin D1 is found to be decreased meanwhile p27 increases and consequently, cells arrested at G1/S phase die either by autophagy or apoptosis (197, 198).

In the specific case of everolimus, it is known that this drug inhibits the aberrant activity of mTOR by inducing arrest at G_1_-phase and sensitizing endothelial and tumoral cells to cisplatin and radiotherapy effects through apoptosis enhancement (197). Such effect occurs due to everolimus ability to block p53-induced p21 expression (201). Everolimus has also been tested in cervical cancer cell lines with a remarkable ability to inactivate efficiently the HPV16 E7 oncoprotein inhibiting cell proliferation (202). The capacity of everolimus-based combinations to inhibit cell proliferation from several cancer types has been reported for breast cancer (203, 204), renal cell carcinoma (205, 206), and thyroid cancer (207) in clinical trials.

In addition to everolimus and temsirolimus, three natural compounds that have been reported as mTOR inhibitors including curcumin, resveratrol and epigallocatechin gallate (EGCG)(208).These compounds proved to be able to induce cytotoxicity in the HeLa cell line when administrated along with radiation. Nevertheless, it is worth to notice that neither everolimus nor temsirolimus seem to be selective for all cancer cell lines as EGCG, resveratrol or curcumin (209). The pro-apoptotic effect of everolimus combined with paclitaxel has been successfully shown for HeLa and SiHa cell lines. In addition, it has been demonstrated that both compounds inhibit the PI3K/AKT/mTOR pathway (210).

Recently, the combination of a daily everolimus dose administrated with standard chemotherapy for newly diagnosed patients with glioblastoma was evaluated in order to determine its efficacy. Even though everolimus has proved to be effective in several published data, it was evident that its efficacy in clinical trials is not as equal than in *in vitro* models. The administrated treatment was not efficient for improving clinical outcomes yet lead to increased toxicity. Moreover, it was suggested that one of the reasons for such lacking of efficacy could be the activation of the Akt pathway due to S6 feedback loop driven by mTORC2 so, it has been proposed that a dual inhibition of mTORC1 and mTORC2 could prevent such Akt activation (211).

An mTOR inhibitor derived from an active fraction of the ethyl acetate extract of *Streptomyces* sp OA293 was reported in 2018. Although it was fully corroborated that such extract lacks any known natural inhibitor of mTOR to date, the metabolite or metabolites present in such active fraction completely inhibited mTORC1 and controlled Akt activation by blocking mTORC2 phosphorylation at Ser2481. Also, this fraction suppressed the activation of 4E-BP1 and P70S6k in cervical cancer cell lines and, induced autophagy and Bax mediated apoptosis. Such extract may represent a better option for improving clinical outcomes in patients once its proved to perform as well as in cell lines (212).

Other rapalogs have been evaluated in clinical trials showing discouraging results in some cases. In 2013, was reported the use of temsirolimus in a phase II study using a dose of 25 mg once a week 4 times. Of 38 patients with cervical cancer enrolled in the study, one of them experienced partial response and 19 had stable disease rendering the effectiveness of temsirolimus alone as questionable (213). According to previous reports performed with cervical cancer cell lines, it was suggested that using mTOR inhibitors could be more efficient when the inhibitors are administrated in combination with other drugs. Three years later, in 2016, Ferreira and colleagues evaluated the maximum-tolerated dose (MTD) of everolimus combined with cisplatin and pelvic radiotherapy, as well as safety and toxicity in 15 patients with advanced stage of cervical cancer in a phase I study. The results showed that although the acceptable dose of everolimus was 5 mg/day, all patients had at least 1 adverse event. Concerning its efficacy, from 12 patients evaluated, 11 showed a complete response, suggesting that 5 mg everolimus together with cisplatin and chemotherapy is a feasible therapy for cervical cancer treatment (207).

Another promising combination using everolimus has been reported in cancer cell lines using metformin, a drug commonly used for diabetes treatment. Metformin induces the inhibition of OXPHOS due to reduced function of respiratory complex I and AMPK activation, which in turn promotes tumor growth reduction through mTOR inhibition, cell cycle arrest and activation of autophagy; therefore, a combination of both drugs could be more successful for cancer treatment. This synergistic effect was evaluated in breast cancer cell lines (MCF-7, MDA-MB-231, and T47D), cultured with a physiological concentration of glucose under hypoxic or normoxic conditions. The obtained results showed that everolimus and metformin cooperate to inhibit mTOR activity, tumor cell growth and colony formation, independently of glucose and O_2_ concentrations (214). A year later, the synergic effect of metformin and rapamycin was evaluated in a pancreatic cancer cell line (SW1990) where a reduced cell proliferation was observed, moreover, cell viability was also reduced when cells were treated with both rapamycin and metformin, importantly, an evaluation of phosphorylated mTOR showed that only a combination of the two drugs was capable to suppress the mTOR pathway. Finally, using a xenograft tumor model, the capacity of metformin and rapamycin to inhibit tumor growth was confirmed (215).

As mentioned before, the use of mTORC1 inhibitors in clinical trials has not been as successfully demonstrated as it has been in cancer cell lines. A possible explanation to this phenomenon could be that upon mTORC1 inhibition, PI3K-AKT cell signaling is stimulated and, consequently it may increase the survival of cancer cells (199). All this because rapamycin and its rapalogs selectively target only mTORC1 without affecting mTORC2, such selective inhibition could prompt feedback loops resulting in AKT activation at ser473 (216). However, it is important to highlight once more that there is plenty of information, which suggests that the use of such inhibitors in combination with other drugs could improve clinical outcome; what is more, inhibiting both mTORC1 and mTORC2 could improve the poor response of other inhibitors observed in clinical trials.

Besides mTORC1 rapalogs, there is another group of mTOR inhibitors known as ATP analogs; such drugs inhibit mTOR kinase activity trough competing with ATP in order to bind to the mTOR kinase domain. ATP donates the phosphate group by which mTOR phosphorylates its target proteins. The ATP analogs inhibit both mTORC1 and mTORC2, interestingly, and because of the resemblance of the kinase domains of mTOR and PI3Ks, this analogs are able to inhibit also PI3K (199).

Inhibition of both PI3K and mTOR ought be effective in eliminating cancer cells. A recent publication tested a low-dose triple drug combination that inhibits the pathways PI3K, Akt and mTOR in seven cell lines derived from ovarian clear cell carcinoma (OCCC). The use of the drugs AZD8055, GDC0941, and selumetinib decreased cell proliferation and significantly reduced tumor growth in two OCCC patient-derived xenograft mice models. The results and lack of adverse effects in the mice show that the combination of these three drugs could validate future clinical tests (217).

CC-223 is a competitive inhibitor of the mTOR that targets mTORC1 and mTORC2, preventing up regulation of Akt phosphorylation, a great advantage, if comparing to the rapalogs. In a phase I Dose-Escalation study, CC-22 was evaluated in twenty-eight patients with advanced cancer. Safety, tolerability, non-tolerated dosage, maximum tolerated dosage (MTD), and preliminary pharmacokinetic profile were evaluated; the reported adverse effects were hyperglycemia, rash, fatigue and mucositis, 45 mg/d was determined as the MTD and an inhibition of phosphorylation of mTORC1/mTORC2 pathway biomarkers present in blood was observed. Taken together these results suggest that CC-223 was tolerable, with manageable toxicities representing a promising antitumor activity compound (218).

Sapanisertib (TAK-228) is a potent and highly selective mTORC1/mTORC2 inhibitor that has been tested in non-hematological malignancies. In this study, sixty-one patients with advanced solid tumors were given daily or a weekly dose of TAK-228 alone or in combination with paclitaxcel. The results showed that just one patient that received TAK-228 plus paclitaxel showed a complete response, moreover, three patients that took TAK-228 plus paclitaxel and two patients with a daily dose of TAK-228 showed a partial response. Additionally, safety analyses showed that fatigue was the main adverse effect, followed bypruritus, lack of appetite and diarrhea, among others but any severe effect related to the treatment was reported. Contrary to everolimus and temsirolimus treatment, anemia and thrombocytopaenia were not reported as adverse effects by consuming TAK-228. Even though the authors emphasize a positive response to TAK-228 alone or in combination with paclitaxel, which could guarantee further investigations, it is only highlighted a positive response for some solid tumors (219).

Recently, specific mTORC1/mTORC2 inhibitors, torin2, INK-128, and NVP-Bez235 (which also inhibits PI2K), were tested on LNT-229 human glioblastoma cells. INK-228 and NVP-Bez235 inhibited the phosphorylation of mTOR targets S6RP and NDRG1, and together with torin2 showed a better capacity of inhibiting mTOR pathway when compared to rapamycin due to a more effective inhibition of 4EBP phosphorylation. The main contribution of this paper was that they highlight the metabolic effects of partial mTOR pathway inhibition by rapamycin and rapalogs to economize resources when cells are exposed to nutrient deficiency and hypoxic conditions, which could promote survival of tumor cells hence, highlighting the use of dual mTORC1/mTORC2 inhibition because such inhibitors are able to target dividing cells more efficiently (220).

Another mTORC1/mTORC2 inhibitor, CC-223, was evaluated in a phase II study including 47 patients with non-pancreatic neuroendocrine tumors. Tolerability, preliminary efficacy and pharmacokinetic of CC-223 was evaluated in a daily dose. The results were consistent with those presented in cell lines; anti-tumor activity was assessed, and the data obtained indicated that the drug was safe for patients (221). Additionally, other mTORC1/mTORC2 known as vistusertib was evaluated in a phase II study for patients with relapsed or refractory diffuse large B cell lymphoma, in this specific case, the dual inhibitor vistusertib did not show any advantage over mTORC1 inhibitors in the group of patients evaluated (222).

The combination of mTOR inhibitors with other drugs or treatments is thought to be more effective than just one treatment alone. Recently, the oral administration of PQR309, a dual PI3K and mTORC1/mTORC2 inhibitor, was evaluated in a phase I trial of patients with advanced solid tumors. The patients presented several adverse effects as fatigue, rash and loss of appetite and partial response was reported (223).

In sum, rapamicyn and rapalogs inhibit mTORC1 as demonstrated in several *in vitro* experiments (160), though incomplete mTOR signaling occurs due to these drugs incapacity of inhibit mTORC2 too, and in consequence, it has been suggested that cancer cells could survive because of Akt activation, for this reason and aiming to replicate the successful results observed in cell lines to patients, it is imperative to evaluate the synergic effect of mTOR inhibitors with other drugs or treatments that have shown promising results in patients and also lead the inhibition of mTOR signaling by drugs to perform a complete inhibition of mTORC1 and mTORC2 in order to guarantee clinical outcome.

## Concluding Remarks

mTORC1 is widely described as an important regulator of cell growth, acting on the regulation of anabolic processes such as the synthesis of proteins, lipids, and the inhibition of autophagy. Importantly, mTORC1 is also involved in the regulation of mitochondrial metabolism and mitochondrial functions. In tumor exists a continuous two-way communication between mitochondria and the nucelus that orchestrates production of the mitochondrial encoded proteins and the nuclear-encoded mitochondria proteins to meet the cells continually changing energy and biosynthetic requirements. mTORC1 plays the major role in the regulation of the mitochondrial protein translation, moreover mTOR is an important regulator of mitochondrial turnover by regulating mitochondrial fusion and fission processes mainly deregulated in cancer and that are associated with chemotherapy resistance.

However, it is necessary to intensify research to clarify the participation of mTORC1 in the regulation of these mitochondrial functions and their impact on the aggressiveness of tumors. The fact that mitochondria promotes metabolic plasticity associated with resistance to therapy and the existence of several drug able regulators, proposes this events as promising therapeutic targets in cancer. In addition to the regulatory actions performed by mTOR in mitochondrial functions it represents an opportunity to deeply study for therapy, developing treatment plans with synergy, mainly using mTOR inhibitors, and mitochondrial inhibitors. In this manner, the use of metformin is an attractive therapeutic option with probed efficacy in clinical trials.

## Author Contributions

KC and AG: conception and design. KC, MT, ES, and AG: wrote and critically review the manuscript. KC, MT, and AG: figure design and elaboration. AG: directed manuscript.

### Conflict of Interest

The authors declare that the research was conducted in the absence of any commercial or financial relationships that could be construed as a potential conflict of interest. The handling editor declared a shared affiliation, though no other collaboration, with with several of the authors AG and KC.
